# Restoration of norepinephrine release, cognitive performance, and dendritic spines by amphetamine in aged rat brain

**DOI:** 10.1111/acel.14087

**Published:** 2024-02-08

**Authors:** Serena Scognamiglio, Yousef M. Aljohani, Thao T. Olson, Patrick A. Forcelli, Ghazaul Dezfuli, Kenneth J. Kellar

**Affiliations:** ^1^ Department of Pharmacology & Physiology Georgetown University Medical Center Washington, DC USA

**Keywords:** aging, amphetamine, dendritic spines, norepinephrine release

## Abstract

Age‐related dysfunctions in specific neurotransmitter systems likely play an important role in cognitive decline even in its most subtle forms. Therefore, preservation or improvement of cognition via augmentation of neurotransmission is a potential therapeutic strategy to prevent further cognitive deficits. Here we identified a particular neuronal vulnerability in the aged Fischer 344 rat brain, an animal model of neurocognitive aging. Specifically, we demonstrated a marked impairment in glutamate‐stimulated release of norepinephrine (NE) in the hippocampus and cerebral cortex of aged rats, and established that this release was mediated by N‐methyl‐d‐aspartate (NMDA) receptors. Further, we also demonstrated that this decrease in NE release is fully rescued by the psychostimulant drug amphetamine (AMPH). Moreover, we showed that AMPH increases dendritic spine maturation, and importantly shows preclinical efficacy in restoring memory deficits in the aged rat through its actions to potentiate NE neurotransmission at β‐adrenergic receptors. Taken together, our results suggest that deficits in glutamate‐stimulated release of NE may contribute to and possibly be a determinant of neuronal vulnerability underlying cognitive decline during aging, and that these deficits can be corrected with currently available drugs. Overall these studies suggest that repurposing of psychostimulants for age‐associated cognitive deficits is a potential avenue to delay or prevent cognitive decline and/or frank dementia later in life.

## Graphical abstract

The aged brain is characterized by decreases in glutamate‐stimulated norepinephrine release in the hippocampus and cerebral cortex that is rescued by the psychostimulant amphetamine (AMPH). From behavioral and morphological studies, AMPH rescues recognition and spatial memory deficits and favors dendritic spine maturation resulting in an overall increase in the density of spine subtypes that are associated with stronger synaptic connections integral to memory function.

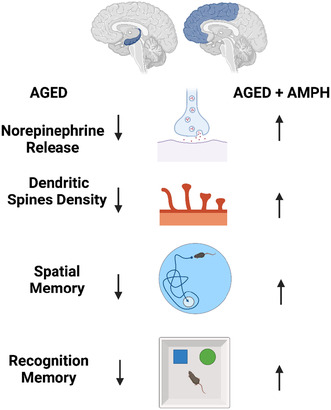

**Abbreviations**
AMPH: amphetamine
CNS: central nervous system
d‐AMPH: d‐amphetamine
DG: dentate gyrus
DI: discrimination index
Glu: glutamate
NE: norepinephrine
NMDA: N‐methyl‐D‐aspartate
NOR: novel object recognition

## 1 INTRODUCTION

Norepinephrine (NE) is an important and widespread central nervous system (CNS) neuromodulator that regulates many critical processes essential to our well‐being, including arousal, sleep–wake cycles, autonomic nervous system, and neuroendocrine functions. NE also plays a role in various aspects of mood and affect, and it has long been a target of some antidepressant drugs. In addition, NE is important in cognitive processes, such as attention, focus, processing of information, response speed, memory consolidation, recall, and plasticity (Aston‐Jones & Cohen, 2005; Breton‐Provencher et al., 2022; Murchison et al., 2004; Thorp et al., 2020). Therefore, disturbances in NE neurotransmission in the CNS can have wide‐ranging and important consequences.

Previous studies found that NE release stimulated by exogenous N‐methyl‐D‐aspartate (NMDA) in the cerebral cortex and hippocampus is significantly decreased in aged rats compared to young rats (Gonzales et al., 1991; Pittaluga et al., 1993). Here we expanded these studies, comparing cerebral cortex and hippocampus from young (2‐ to 4‐month‐old) and aged (18‐ to 24‐month‐old) Fischer 344 rats, an established model of aging (Gallagher et al., 2011). We measured glutamate (Glu)‐stimulated release of [^3^H]‐NE in slices and confirmed that there is an age‐related decrease in this Glu‐stimulated [^3^H]‐NE release that is largely mediated by NMDA receptors as compared to alpha‐amino‐3‐hydroxy‐5‐methyl‐4‐isoxazole propionate receptors, which have also been implicated in NE release (Malva et al., 1994). Importantly, we also determined whether and to what extent the age‐related decrease in Glu‐stimulated [^3^H]‐NE release can be rescued, that is restored, by the psychostimulant drug d‐amphetamine (d‐AMPH). In addition, to determine whether the age‐related deficit in Glu‐stimulated NE release in aged rats affected cognition, we measured performance in two cognitive tasks in young and aged rats before and after acute and chronic treatment with AMPH, and determined whether the effects of AMPH on cognitive performance were dependent on its actions via NE. Finally, we examined how aging and chronic AMPH affect a synaptic component related to cognition by evaluating dendritic spine density and morphology in the rat cortex and hippocampus.

## 2 RESULTS

### 2.1 Glutamate‐stimulated [^3^H]‐NE release is decreased in the cortex and hippocampus from aged rats

**FIGURE 1:**
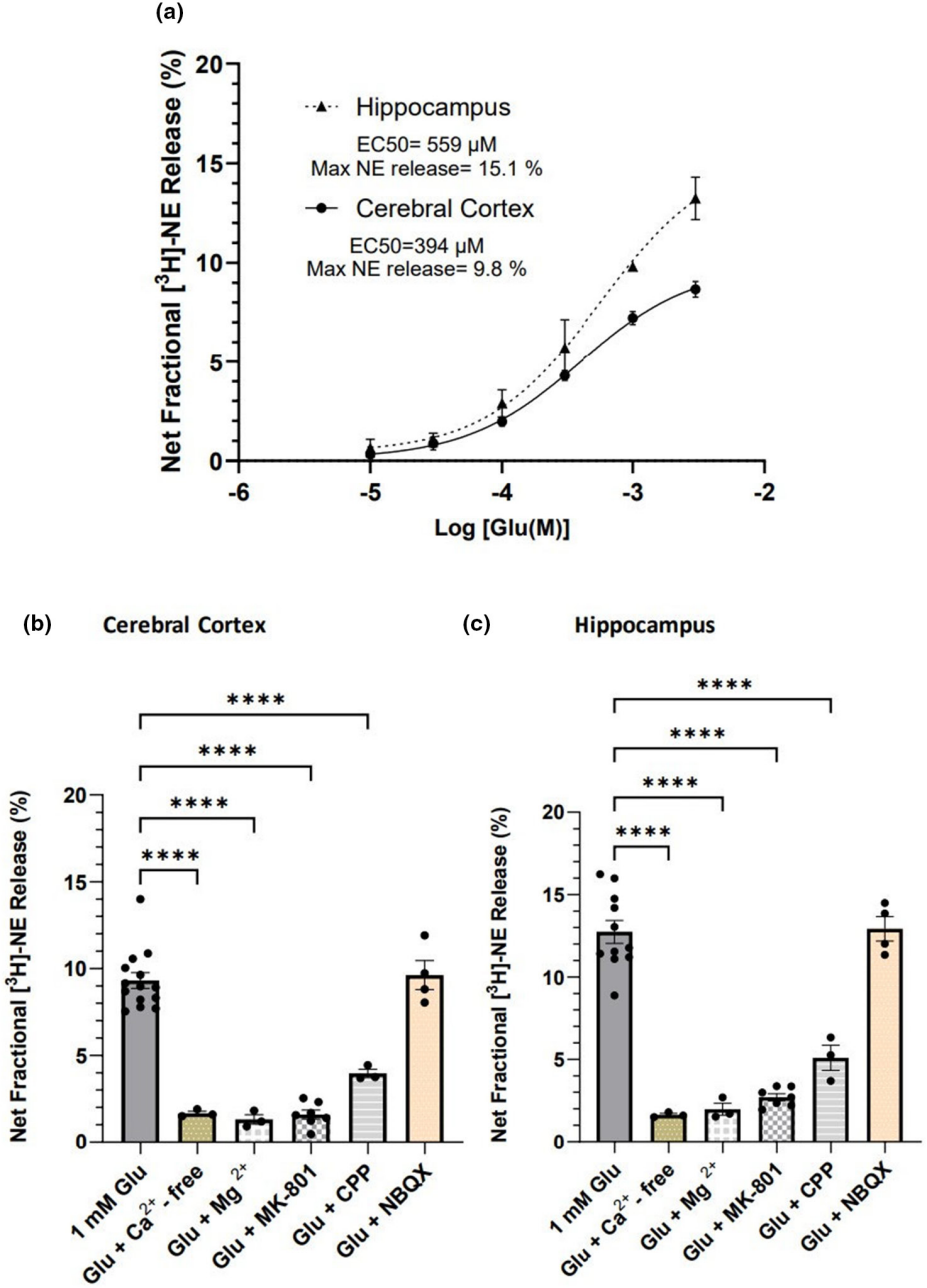
Characteristics of glutamate‐stimulated [^3^H]‐norepinephrine ([^3^H]‐NE) release in rat brain slices. (a) The concentration–response curve of glutamate‐stimulated [^3^H]‐NE release in cerebral cortical (solid line) and hippocampal (dotted line) tissue slices from young rats. Each point represents the mean ± SEM of three (cortex) or two (hippocampus) experiments. (b, c) 1 mM Glu stimulated [^3^H]‐NE release in cerebral cortical (b) and hippocampal (c) tissue slices from young (2‐ to 3‐month‐old) Fischer 344 rats either in the absence of added Ca^+2^ or in the presence of Mg^+2^ (1.2 mM), MK‐801 (10 μM), CPP (100 μM) or NBQX (10 μM), as indicated. Each bar represents the mean ± SEM of net fractional release across individual experiments. ****p < 0.0001.

Glu‐stimulated release of [^3^H]‐NE from cerebral cortex and hippocampus from young rats is concentration‐dependent, with EC_50_ values of 394 μM [95% CL: 284 μM, 579 μM] and 559 μM [CL: 291 μM, 1299 μM], respectively (Figure 1a). The maximum net fractional Glu‐stimulated release of [^3^H]‐NE release for these two brain areas were 9.8% of tissue content [95% CL: 9.0%, 10.8%] and 15.1% [95% CL: 12.9%, 19.6%], respectively (Figure 1a). These studies indicate the maximum Glu‐stimulated release of [^3^H]‐NE is higher in the hippocampus than in the cortex. Glu‐stimulated [^3^H]‐NE release was robust in both the cortex and the hippocampus, with [^3^H]‐NE release stimulated by 1 mM Glu being five to seven times greater than basal release, which was typically 1.5% of total [^3^H]‐NE content.

To identify the glutamate receptor subtype that mediates this Glu‐stimulated [^3^H]‐NE release from the cortex and the hippocampus, we examined its physiological and pharmacological characteristics. A mixed effects analysis revealed a significant effect of pharmacological treatment on Glu‐stimulated cortical release of [^3^H]‐NE [*F*_(5,15)_ = 174.3] (Figure 1b) and Glu‐stimulated hippocampal release of [^3^H]‐NE [*F*_(5,15)_ = 98.95] (Figure 1c). In both brain regions, [^3^H]‐NE release was reduced by more than 80% when calcium was omitted from the buffer solution, consistent with a physiological calcium‐dependent release mechanism. Importantly, the release is also markedly reduced in the presence of 1.2 mM magnesium (*p* < 0.001, Dunnett's multiple comparison tests), which blocks NMDA receptor channels, and, consistent with this, MK‐801, an NMDA receptor channel blocker and CPP, an NMDA receptor competitive antagonist, both blocked Glu‐stimulated [^3^H]‐NE release (*p* < 0.001). In contrast, NBQX, an AMPA receptor antagonist, failed to inhibit release. This Glu‐stimulated [^3^H]‐NE release profile in both the cortex and the hippocampus indicates that the release is mediated predominantly by NMDA receptors.

**FIGURE 2:**
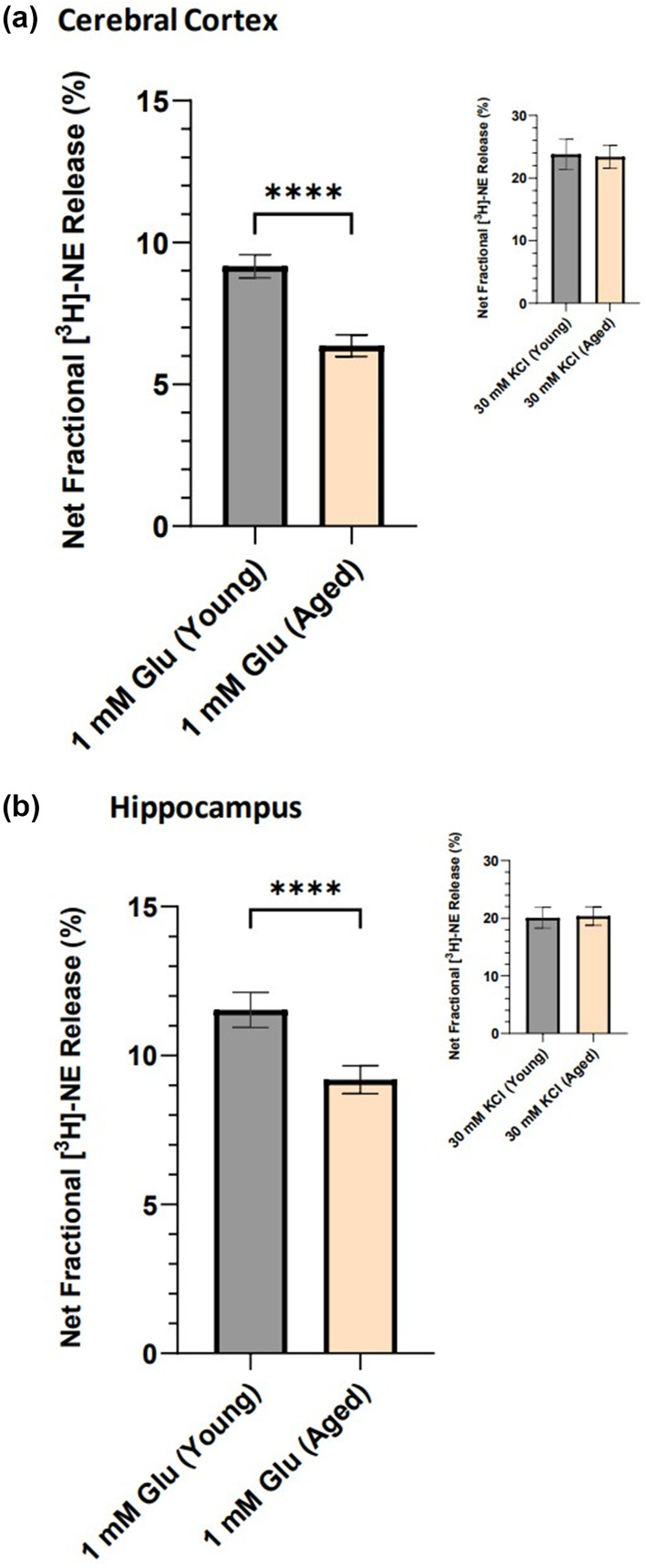
Glutamate‐stimulated [^3^H]‐norepinephrine ([^3^H]‐NE) release is decreased in cortical and hippocampal tissues slices from aged Fischer 344 rats. (a) [^3^H]‐NE release stimulated by 1 mM glutamate in cortical slices from young (2‐ to 3‐month‐old; *n* = 31) versus aged (18‐ to 24‐month‐old; *n* = 21) rats, or by 30 mM K^+^ in young (*n* = 11) versus aged (*n* = 11) rats (inset). (b) [^3^H]‐NE release stimulated by 1 mM glutamate in hippocampal slices from young (*n* = 18) versus aged (*n* = 18) rats, or by 30 mM K^+^ in young (*n* = 11) versus aged (*n* = 11) rats (inset). Each bar is the mean ± SEM of net fractional release in each group. ****p < 0.0001.

Importantly, as shown in Figure 2a,b, [^3^H]‐NE release stimulated by 1 mM glutamate was significantly lower in both the cortex and the hippocampus from aged rats compared to release from young rats (one‐sample t test, *p* < 0.0001). In contrast, [^3^H]‐NE release stimulated by a depolarizing concentration of potassium (30 mM) was nearly identical from young and aged rats in both of these brain areas (Figure 2a,b insets). This age‐related decrease in NMDA receptor‐mediated Glu‐stimulated [^3^H]‐NE release in rat cortex and hippocampus agrees with earlier reports of an age‐related decrease in [^3^H]‐NE release evoked by the agonist NMDA, itself (Gonzales et al., 1991; Pittaluga et al., 1993).

### 2.2 Restoration of age‐related decrease in Glu‐stimulated [^3^H]‐NE release by AMPH

**FIGURE 3:**
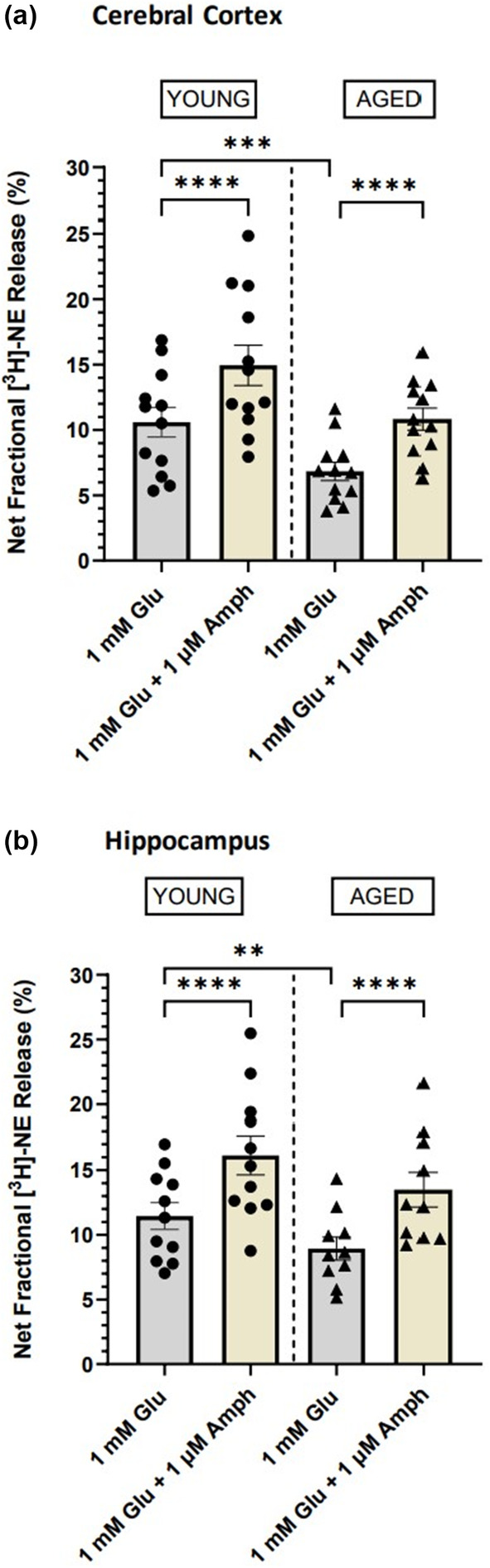
d‐Amphetamine sulfate restores the deficit in glutamate‐stimulated release of [^3^H]norepinephrine ([^3^H]NE) in the aged Fischer 344 rat brain. [^3^H]‐NE release in cerebral cortical and hippocampal slices from young (2‐ to 3‐month‐old; *n* = 11–12) and aged (18‐ to 24‐month‐old; *n* = 10–12) rats in the absence and presence of 1 μM d‐amphetamine sulfate (a, b). Data are expressed as mean ± SEM of % net fractional release for the number of points shown. **p < 0.01, ***p < 0.001, ****p < 0.0001.

To determine whether the decreased Glu‐stimulated [^3^H]‐NE release found in aged rats can be restored to levels of release found in young rats, we compared release from young and aged rats in the absence and presence of AMPH, a psychostimulant drug that increases monoamine neurotransmission through several different mechanisms (Sulzer et al., 2005). After examining the concentration–response relationship of d‐AMPH to potentiate release in the presence of 1 mM Glu (Figure S1), we chose a concentration of 1 μM AMPH to use in these studies. As shown in Figure 3a, a two‐way ANOVA revealed a significant main effect of age [*F*_(11,33)_ = 14.27; *p* < 0.0001] and drug treatment [*F*_(3,33)_ = 38.94; *p* < 0.0001] demonstrating that 1 μM AMPH increased Glu‐stimulated release of [^3^H]‐NE in the cortex. Similarly, in the hippocampus (Figure 3b), a two‐way ANOVA also revealed a significant main effect of age [*F*_(10,28)_ = 20.77; *p* < 0.001] and drug [*F*_(3,28)_ = 39.74; *p* < 0.001] demonstrating that the deficit in hippocampal Glu‐stimulated [^3^H]‐NE release was also rescued by AMPH. Of particular importance for these studies, AMPH restored the decreased [^3^H]‐NE release in both of these brain areas from the aged rats to the release levels seen in the young rats measured in the absence of drug (Figure 3).

### 2.3 Decreased cognitive functions in aged rats is ameliorated by AMPH treatment

To determine whether the age‐related decrease in Glu‐stimulated NE release is associated with a change in cognitive performance, we examined young and aged rats in two kinds of cognitive tasks: The novel object recognition (NOR) task, which assesses recognition memory (Ennaceur & Delacour, 1988; Sivakumaran et al., 2018) where age‐related deficits have been established (Burke et al., 2010; Ennaceur & Delacour, 1988; Sivakumaran et al., 2018) and the Barnes maze, which measures hippocampal based‐spatial learning and memory (Bach et al., 1999; Barnes, 1979; Weber et al., 2015).

### 2.4 The NOR task

**FIGURE 4:**
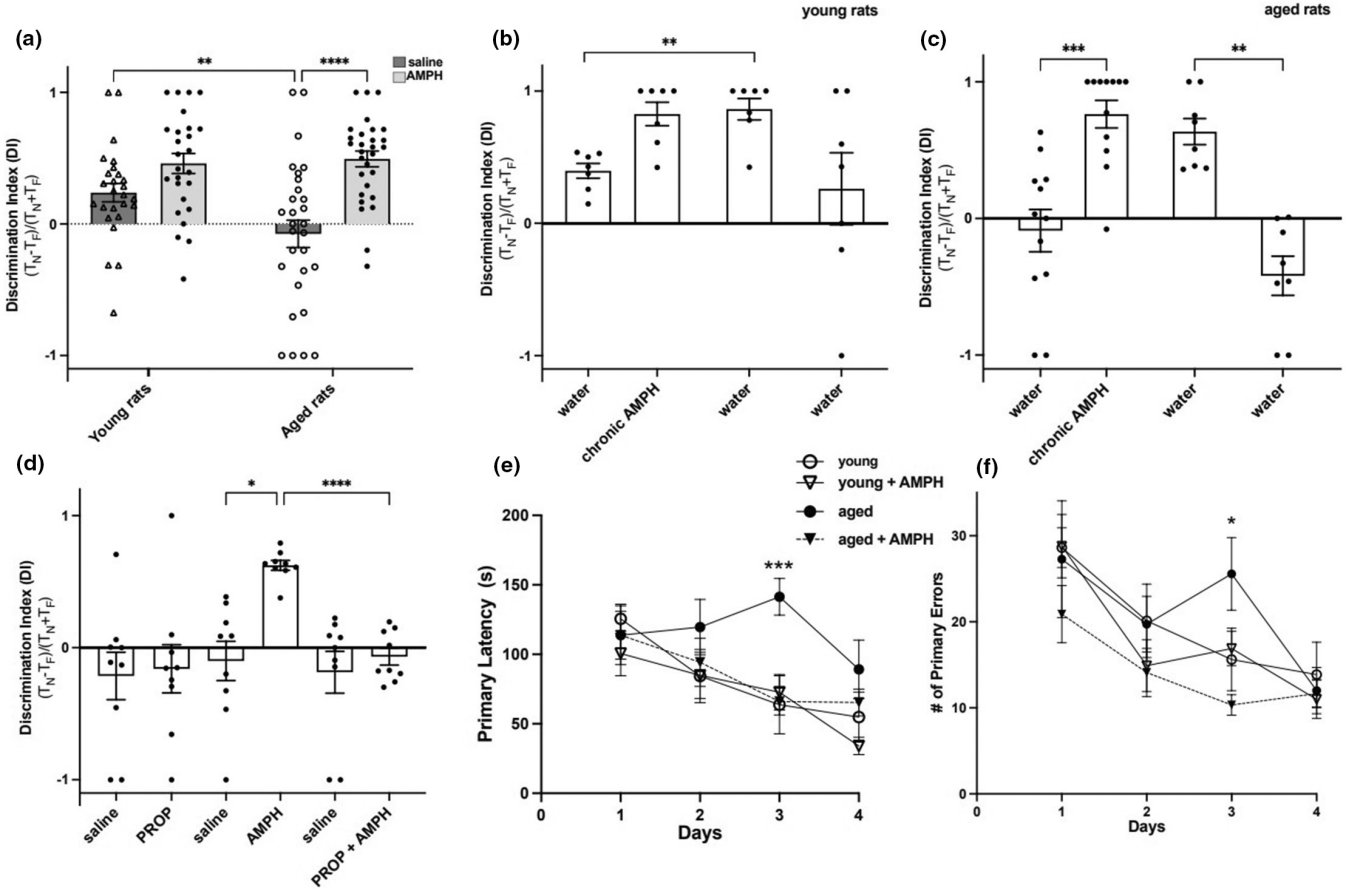
Amphetamine enhances 24‐h object recognition and spatial memory in aged Fisher 344 rats. (a) A single IP injection of amphetamine (AMPH; 0.5 mg/kg) administered 20 min before the test phase does not significantly affect object recognition memory in young rats, but does significantly enhance object recognition memory in aged rats. (b, c) AMPH administered for 2 weeks via the drinking water enhances object recognition memory in both young and aged rats, and this enhanced memory performance is still seen 2 weeks but not 10 weeks after the administration of amphetamine is stopped. (d) A single low‐dose injection of propranolol (0.5 mg/kg) does not in itself affect memory performance on the NOR task in aged rats, but pretreatment with this dose of propranolol administered 40 min prior to an IP injection of AMPH (i.e., 60 min before the NOR test phase) reverses the AMPH effect on object recognition in aged rats. *p < 0.05; **p < 0.01; *** *p* < 0.001; ****p < 0.0001 compared with the corresponding control groups. (e, f) Chronic AMPH improves spatial memory in aged rats in a Barnes maze task by reducing both primary latency time (e) and number of errors (f). *p < 0.05; ***p < 0.001. *n* = 26–29/group (NOR) and 7–10/group (Barnes maze).

Following training and a delay interval of 24 h, which assesses long‐term recognition memory (Antunes & Biala, 2012), the effect of a single IP injection of 0.5 mg/kg AMPH administered 20 min before the NOR test phase was evaluated in young (*n* = 26) and aged (*n* = 29) rats. The discrimination index (DI), a metric of preferential exploration of new versus old object, was measured at 1 min into the test phase, which previously has been found to be the period most sensitive for NOR assessment of memory (Dix & Aggleton, 1999; Sawangjit et al., 2018). A one‐sample t test was used to compare performance to chance levels (DI = 0). A mixed effects analysis revealed a significant age × treatment interaction on the NOR task, as measured by the DI [*F*(_1,106_) = 4.762, *p* = 0.03] (Figure 4a). Young rats treated with saline showed the expected preference for the novel object with a DI = 0.238 ± 0.070 (one‐sample t test; *t* = 3.4, df = 25, *p* = 0.002), and their performance after acute treatment with AMPH did not improve significantly. In contrast to the young rats, the DI in aged rats treated with saline did not differ significantly from chance performance (Figure 4a), but after acute treatment with AMPH, the DI in aged rats increased to 0.49 ± 0.06, which was significantly higher than performance at baseline (*t* = 8.2, df = 28, *p* < 0.0001), and approximately the same as in young rats.

We next investigated whether chronic administration of AMPH would improve object recognition memory, and whether this effect would persist after drug cessation. As shown in Figure 4b,c, respectively, a baseline DI was measured in young and aged rats drinking water only, followed by treatment with AMPH via their water for 2 weeks (0.25 mg/kg during Week 1 and 0.5 mg/kg during Week 2). The DI was then measured again at the end of the 2‐week period on AMPH, after which the rats were switched back to water, and the DI was then measured again 2 and 10 weeks later. Consistent with the acute studies, the young rats (Figure 4b) exhibited the expected preference for novel objects, with a DI of 0.39 ± 0.05 (one sample t test, *t* = 7.10 df = 6; *p* = 0.0004), while the DI for the aged rats at baseline prior to drug treatment (Figure 4c) did not differ significantly from chance. In young rats (Figure 4b), a one‐way ANOVA did not reveal a significant effect of drug treatment across all groups [*F*_(1.274,7.643)_ = 3.965, *p* = 0.077], probably because the baseline scores in these young rats were already above chance. However, Tukey's post hoc test revealed that chronic administration of AMPH for 2 weeks resulted in significantly higher DI scores (*p* < 0.01); importantly, the DI scores were still significantly higher than baseline 2 weeks after being switched back to water (*p* < 0.01), but not after 10 weeks on water.

In aged rats also (Figure 4c), a mixed‐effect analysis revealed a significant effect of chronic AMPH treatment on NOR performance [*F*_(2.272,18.94)_ = 25.64, *p* < 0.0001]. Thus, in aged rats treated chronically with AMPH, the DI scores (0.76 ± 0.1) was significantly higher than the baseline score (−0.08 ± 0.1; *p* = 0.0009). Moreover, 2 weeks after the aged rats were switched back to water, their mean DI score (0.63 ± 0.09) remained higher than baseline, and in fact did not differ significantly from their score while they were still on the drug, suggesting that chronic AMPH treatment in aged rats produced a relatively long‐lasting effect on performance in the NOR task. However, 10 weeks after cessation of the AMPH treatment, the aged rats no longer displayed a preference for the novel object; in fact, interestingly, rather than reflecting chance, their DI scores (−0.4 ± 0.14; *p* = 0.02) suggested a preference for the familiar object (Figure 4c), which could suggest an improved memory for the familiar object under these conditions.

### 2.5 NE mediates performance in the NOR task via β‐adrenergic receptors

To determine whether NE signaling per se mediates AMPH's effect on performance in the NOR task, we pretreated rats with propranolol, a potent beta‐adrenergic receptor antagonist (Baker, 2005), before testing them in the NOR task. Since previous studies indicated that higher doses of propranolol itself can affect memory (Cahill et al., 1994; Villain et al., 2016), we used a low dose (0.5 mg/kg), which alone did not affect baseline performance in the NOR task (Figure 4d). Consistent with our previous studies shown in Figure 4a, a single injection of AMPH administered 20 min before the NOR task improved performance as measured by the DI [*F*_(3.052,24.41)_ = 6.123; *p* = 0.002] (Figure 4d). Importantly, as also shown in Figure 4d, propranolol injected 40 min prior to AMPH blocked the improvement in the NOR task seen with AMPH alone (*p* < 0.0001).

### 2.6 The Barnes maze task

To further assess cognitive function and the effect of AMPH in aged rats, we employed the Barnes maze, a hippocampal‐based spatial learning and memory task. Four treatment groups were compared: young rats on normal drinking water (*n* = 8), young rats administered chronic AMPH (*n* = 10), aged rats on normal drinking water (*n* = 7), and aged rats administered chronic AMPH (*n* = 9). A comparison of the mean of four trials per animal per day assessed by a three‐way repeated measures ANOVA revealed a significant day *×* age (aged vs. young) *×* treatment (water vs. chronic AMPH) interaction effect [*F*_(3,90)_ = 3.08, *p* = 0.03]. Specifically, pairwise comparisons with (Sidak corrections) indicated that by the third day of the acquisition phase (Figure 4e), aged rats on normal drinking water displayed a deficit in spatial memory compared to young rats on normal drinking water, as indicated by an increase in their primary latency to find the escape hole (*p* = 0.035). In contrast, aged rats chronically treated with AMPH displayed a significant decrease in primary latency compared to the aged rats treated with normal drinking water only (*p* = 0.003). In fact, the primary latencies of the aged rats chronically treated with AMPH were similar to the latencies in young rats (Figure 4e). We also compared the number of primary errors across blocks comprised of four sessions (each) and found a significant effect of treatment on the error rate [*F*_(1,30)_ = 6.34, *p* = 0.017], as analyzed by a three‐way repeated measures ANOVA. Aged rats on chronic AMPH showed a decrease in errors by Day 3 in comparison with aged rats on normal drinking water (*p* = 0.049), following which performance leveled off and no further improvement was measurable (Figure 4f).

### 2.7 Chronic AMPH treatment in aged Fischer 344 rats alters dendritic spine distribution and morphology

**FIGURE 5:**
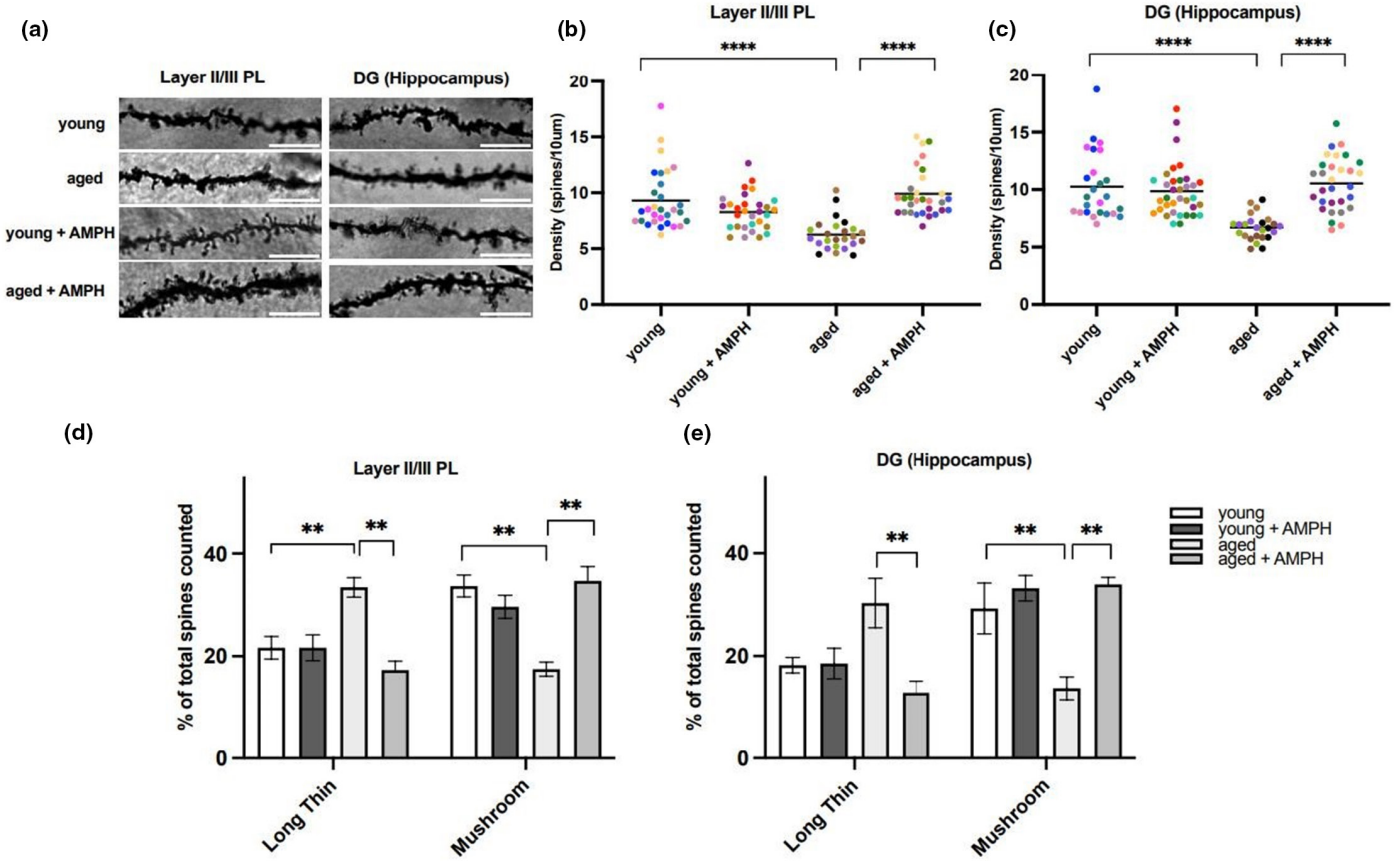
Chronic amphetamine treatment augments dendritic spine density and favors a morphological shift toward mature spines in aged Fischer 344 rats. (a) Representative example of Golgi staining of basal dendrites in layers II/III of the prelimbic cortex (PL) and in the hippocampal dentate gyrus (DG) of control and amphetamine (AMPH)‐treated groups. (b, c) Spine density in layer II/III of the PL and in the DG of the hippocampus. Each individual point represents the mean of three randomized dendritic segments from each rat for density analyses (*n* = 5–6 rats per group). Each animal is represented by a different color. ****p < 0.0001. (d, e) Analysis of shifts in the relative abundance of long thin and mushroom spines (classified as described in Section 2) after AMPH treatment in layer II/III of the PL (d) and in the DG of the hippocampus (e) **p < 0.01 compared with the corresponding vehicle control groups and young versus aged.

Structural plasticity of dendritic spines in the form of density and morphological changes are important to the neurobiology of memory and learning (Li et al., 2004; Morris & Hebb, 1999); therefore, we compared spine density and morphology in the prelimbic cortex and hippocampal dentate gyrus (DG) from a new cohort of young and aged rats not previously tested on any behavioral tests (Figure 5a–e). To do this, all the animals were given either the normal drinking water or water with AMPH to determine whether aging and/or chronic treatment with AMPH affected those parameters. The prelimbic cortex and hippocampal DG were chosen because both areas are included in the slice preparation used in the release assays previously discussed and both areas are important to cognition. In the prelimbic cortex (Figure 5b), a mixed model analysis revealed a significant interaction between age and drug treatment [*F*_(1,99.677)_ = 35.022, *p* = 4.627e‐8]. Pairwise comparisons (with Sidak corrections) further revealed that cortical spine density was significantly reduced in untreated aged rats compared to the density in untreated young animals (*p* = 1.694e‐6). Comparisons of the two AMPH‐treated groups with the untreated groups revealed that the drug increased cortical spine density significantly in aged rats (*p* = 6.541e‐10), resulting in a spine density in aged rats that was similar to the density in the young rats. In contrast, AMPH did not increase spine density in the young rats (Figure 5b).

Similarly, in the hippocampus (Figure 5c), a mixed model analysis revealed a significant main effect of age [*F*_(1,88.885)_ = 16.021, *p* = 1.30e‐4], and a significant interaction between age and drug [*F*_(1,88.885)_ = 25.591, *p* = 2.25e‐6]. As in the cortex, pairwise comparisons (with Sidak corrections) revealed that hippocampal spine density was significantly reduced in untreated aged rats compared to untreated young rats (*p* = 2.317e‐8). Comparisons between the AMPH treatment groups with the untreated control groups revealed that AMPH increased spine density significantly in the aged rats (*p* = 1.359e‐9) but not in the young rats.

Morphological analyses of spines in the prelimbic cortex (Figure 5d) revealed that AMPH treatment induces spine remodeling characterized by a decrease in long thin (immature/transient) spines and an increase in mushroom (stable mature) spines in aged rats. A three‐way ANOVA (spine subtype *×* age *×* treatment) analyzing the percentage of spines within each experimental group indicated a significant main effect between spine subtype *×* age interaction [*F*_(1,38)_ = 8.839; *p* = 0.0051] and significant main effect between all three factors (subtype *×* age *×* drug) [*F*_(1,38)_ = 35.82; *p* < 0.0001]. Post hoc comparisons indicated that in comparison with young rats, aged rats displayed a significant decrease in mushroom spines (*p* = 0.0012) concomitant with an upregulation in long thin immature spines (*p* = 0.004). These findings are consistent with previous studies demonstrating structural reorganization occurring in normal aging (Dickstein et al., 2007). Importantly, chronic AMPH upregulated the number of mushroom spines in comparison with untreated aged controls (*p* = 0.0012), a phenomenon that was not observed in the young rats.

Similarly, in the hippocampus (Figure 5e), a three‐way ANOVA analyzing spine distribution revealed a significant effect between spine subtype and age [*F*_(1,20)_ = 5.73; *p* = 0.0266] and a significant main effect between all three factors [spine subtype × drug × age; *F*_(1,16)_ = 17.19; *p* = 0.0008]. Similar to the findings in the prelimbic cortex, post hoc comparisons revealed that untreated aged animals displayed a decrease in mushroom spine density compared to young controls (*p* = 0.0084) that is rescued by AMPH treatment (*p* = 0.0012). Together, these data indicate that the unfavorable loss of dendritic mushroom spines associated with normal aging can be improved by AMPH treatment (Figure 5e and Figure S2).

## 3 DISCUSSION

Aging exacts a toll on virtually all physiological functions, but for most people a decline in cognitive function and performance is of greatest concern. Brain aging is a natural process involving structural and functional changes that lead to cognitive decline even in the absence of known CNS disease. Thus, therapeutic approaches for improving cognitive function in the aging brain are critically important as the population ages. In Fischer 344 rats, a widely used animal model for aging (Gallagher et al., 2011), we demonstrate an age‐related decrease in Glu‐stimulated release of NE in the cerebral cortex and hippocampus and a concomitant decline in cognitive performance. Our results reveal that NMDA receptor‐mediated NE release in the cerebral cortex and hippocampus is compromised in the aging brain independent of any known pathologies. In contrast to the significant age‐related decrease in Glu‐stimulated NE release, release stimulated by a depolarizing concentration of K^+^ was not decreased in the aged rat brain. This indicates that aging does not directly affect the neuronal release mechanism but instead suggests that NMDA receptors, rather than release dynamics, are compromised in aging, as previously suggested (Newcomer et al., 2000). Related to this, Barnes et al. (2000) conducted a study to address the question of whether the reduced NMDA receptor response in aged Fischer 344 rats translates into an altered threshold or absolute magnitude for long‐term potentiation (LTP) induction in the hippocampus. They demonstrated that aged rats in fact show an increase in the threshold for induction of NMDA‐mediated LTP at the perforant path‐granule synapse of the hippocampus.

Our in vitro NE release studies reveal selective vulnerabilities of NMDA receptor‐mediated release during brain aging. Specifically, we demonstrate that this NMDA receptor‐mediated release of NE in cortical and hippocampal regions in the aged rat brain is compromised independent of any pathologies. Our data are consistent with studies demonstrating that NMDA receptor‐mediated neurotransmission decreases with age (Gonzales et al., 1991; Pittaluga et al., 1993) and probably contributes to cognitive decline (Newcomer et al., 2000). Although our data indicate that Glu‐stimulated NE release in our model is mediated primarily by NMDA receptors, we cannot rule out a minor contribution by AMPA receptors in mediating the Glu‐stimulated release of [^3^H]‐NE from the Fischer 344 rat brain. In addition, it should be noted that differential effects of glutamate on NE release could also be dependent to some extent on the rat strain (Howells & Russell, 2008).

Importantly, and with clear potential therapeutic implications for addressing problems associated with cognitive decline, our results demonstrate that the age‐related decrease in Glu‐stimulated NE release can be rescued and restored to the levels seen in the young controls by the addition of the CNS stimulant AMPH. AMPH increases synaptic NE via at least two mechanisms, including inhibition of NE reuptake and increasing its release (for reviews, see Faraone, 2018; Sulzer et al., 2005).

The therapeutic potential for improving age‐related cognitive decline by pharmacologically potentiating NE release with AMPH was tested in behavioral studies. Aged Fischer 344 rats, compared to young cohorts, display cognitive deficits in recognition memory, as measured in the NOR task, and in spatial memory, as assessed in the Barnes maze. Our finding that both acute and chronic treatment with AMPH improved performance in both the NOR task and in the Barnes maze further supports the possibility of using AMPH or AMPH‐type drugs to counter cognitive deficits in the aging brain, and possibly even in some identified CNS disorders, as well.

In addition to NE, AMPH is well known to also increase synaptic dopamine, and perhaps other neurotransmitters, but our demonstration that propranolol, a drug that potently blocks β‐adrenergic receptors, reverses the improved cognitive performance induced by AMPH strongly implicates NE in the improved cognitive performance. Furthermore, while NE, the endogenous neurotransmitter at virtually all CNS adrenergic synapses, is a potent agonist at β1‐adrenergic receptors, it is less potent at β2‐adrenergic receptors (Baker, 2005). Together, these data suggest the involvement of NE acting at β1‐adrenergic synapses in the cognitive effects of AMPH. These studies obviously raise the possibility of AMPH as a feasible treatment option for gradual age‐associated cognitive decline among healthy individuals via potentiation of vital NE signaling. Historically, clinical studies of AMPHs have focused on their utility in conditions such as narcolepsy, attention‐deficit/hyperactivity disorder, and depression (for reviews see Bagot & Kaminer, 2013; Stotz et al., 1999; Turner, 2019). Fewer studies have focused on AMPH in the context of aging independent of known pathology or underlying clinical conditions (Garrett et al., 2015; Sassi et al., 2019). AMPH has been safely used chronically in children and adults, including older adult populations, for more than 50 years; thus, our studies presented here may offer the potential for an important treatment option to preserve or improve cognition during aging (Christie et al., 2017; McMaster et al., 2018). However, AMPH's effects on the autonomic nervous system (Idrees et al., 2023) and particularly the cardiovascular system (Sassi et al., 2019) require that such an approach to age‐related cognitive decline be taken with regard to the cardiovascular condition of the recipient and also with regard to other drugs being used concurrently.

The complex mechanisms underlying memory processes are assumed to involve synaptic plasticity, especially in the hippocampus and the cerebral cortex (Kennedy, 2016). The recovery of indices of cognitive performance (memory function) in the aged Fischer 344 rats by AMPH treatment was accompanied by improvements in the density and morphology of dendritic spines, the structural correlates underpinning memory functions. Our results show that chronic AMPH treatment in aged rats leads to dendritic spine remodeling that favors maturation or “rescue” of mushroom spines from immature, long thin spines, resulting in an overall increase in the density of spine subtypes that are associated with stronger synaptic connections integral to memory function (Mahmmoud et al., 2015).

Taken together, our observations suggest that mechanistically the improvements in recognition and spatial memory induced by AMPH in the aging brain could result from restoration of NE release and improvements in spine density and morphology. In summary, our study provides mechanistic insights into the efficacy of AMPH to ameliorate some age‐related neuronal vulnerabilities in the CNS, namely the release of NE in the hippocampus and the cortex by glutamate, and subsequent improvements in cognitive measures. Importantly, these improvements are accompanied by morphological alterations associated with synaptic plasticity. These studies support the need for further investigations into AMPH and related drugs for age‐related cognitive decline.

## 4 MATERIALS AND METHODS

See Table 1.

**TABLE 1:** Key resources table.

Reagent or resource	Source	Identifier
Chemicals, peptides, and recombinant proteins		
[3H]‐Norepinephrine	Perkin Elmer	NET377
d‐Amphetamine sulfate	Sigma‐Aldrich	Cat#1180004
(±)‐Propanolol hydrochloride	Sigma‐Aldrich	Cat#P0884
Hemo‐DE xylene substitute	Electron Microscopy Sciences Inc.	Cat#23412‐01
Permount resinous media	Fisher Scientific, Inc.	Cat#SP15‐100
Ethanol	Fisher Scientific, Inc.	Cat#A4094
L‐glutamic acid	Sigma	Cat#G1251
Calcium chloride dihydrate	Fisher Scientific, Inc.	Cat#C69‐500
Magnesium chloride	Fisher Scientific, Inc.	Cat#M33‐500
MK‐801	Tocris	Cat#0924
CPP	Tocris	Cat#0173
NBQX	Tocris	Cat#1044
Potassium chloride	Fisher Scientific, Inc.	Cat#P217‐500
Critical commercial assays		
FD rapid golgiStain kit	FD NeuroTechnologies, Inc.	Cat#PK401
Experimental models: Organisms/strains		
Rat: Fischer (CDF) Rat	Charles River	Strain 002
Rat: Fisher (CDF) (Rat 18‐to 22‐Month‐Old)	NIA Aging Colony at Charles River	Strain 002
Software and algorithms		
SPSS statistics	IBM	Version 29
Graphpad prism	Dotmatics	Version 9.0
LAS X Lie Science Microscope Software	Leica	Version 1.4.5
BioRender	Science Suite Inc.	RRID: SCR_018361

### 4.1 Resource availability

#### 4.1.1 Lead contact

Further information and requests for resources and reagents should be directed to and will be fulfilled by the lead contact, Ghazaul Dezfuli (gd96@georgetown.edu).

### 4.2 Material availability

This paper does not report any newly generated materials.

### 4.3 Experimental model

All experiments were performed on aged male Fischer 344 rats (18‐ to 22‐month‐old) that were obtained from the NIA breeding facility at Charles River Laboratories (USA). Young male Fischer 344 rats (2‐ to 4‐month‐old) were purchased from Charles River Laboratories (USA). Animals were group‐housed on a 12 h light–dark schedule with ad libitum access to food and water. All experiments were approved by the Georgetown University Animal Care, and Use Committee and adhered to NIH ethical guidelines.

### 4.4 Drugs and reagents

[^3^H]‐NE (11.8 Ci/mmol; PerkinElmer; Shelton, CT, USA). d‐AMPH sulfate and (±)‐propranolol hydrochloride were obtained from Sigma‐Aldrich; all other drugs were from Tocris Bioscience (USA). For the acute behavioral studies, d‐AMPH sulfate and (±)‐propranolol hydrochloride were dissolved in sterile saline and injected intraperitoneally (IP) at doses of 0.5 mg/kg. In the chronic studies, d‐AMPH sulfate was dissolved in drinking water and given at a dose of 0.25 mg/kg/day the first week and 0.5 mg/kg/day the second week to avoid drug tolerance considering the approximate typical water consumption rate/body weight for a rat.

### 4.5 Neurotransmitter release experiments

The animals were sacrificed by decapitation under light isoflurane anesthesia. Brain regions of interest were rapidly dissected and chopped in two directions at approximately 200‐μm thickness using a McIlwain tissue chopper (The Mickie Laboratory Engineering Co., Gomshall, England). The mini tissue slices were then gently dispersed with a pipette in 10 mL oxygenated (95% O_2_/5% CO_2_) Krebs buffer comprised of the following: 118 mM NaCl, 5 mM KCl, 2 mM KH_2_PO_4_, 24 mM NaHPO_3_, 2.5 mM CaCI_2_ 11 mM D‐glucose, 0.2 mM L‐ascorbic acid, 25 mM HEPES 25 (pH 7.4), and placed on an orbital shaker for 5 min. After the washing period, the buffer was aspirated, and the mini tissue slices were loaded with 2 mL [^3^H]‐NE (final concentration 100 nM) in the Krebs buffer and incubated at 37°C shaking water bath for 25 min. After incubation, the tissue slices were washed twice in 10 mL Krebs buffer to remove extracellular [^3^H]‐NE and resuspended in Krebs buffer to 1 mg/μL final concentration. The assay was carried out in a set of 12‐well tissue culture plates. Twenty‐microliter aliquots of standard tissue mini slices (~20 mg of tissue) were placed in nylon mesh baskets. The tissue containing baskets were incubated in a series of 2‐min intervals until a stable baseline release of [^3^H]‐NE was achieved. Drugs were included in three successive baseline samples proceeding the stimulus condition. A 15‐s wash interval followed the stimulus condition to remove any residual stimulus effect followed by three washes at 2 min each to reestablish the baseline release. The 2‐min time period was chosen based on preliminary experiments indicating that it produced the optimal stimulated to basal release ratio. The tissue was then exposed for 2 min to buffer containing a depolarizing concentration of K^+^ (30 mM) before being lysed for 30 min in 0.1 N NaOH to assess the tissue's remaining [^3^H]‐NE content. In some experiments, MgSO_4_ (1.2 mM) was added to, or CaCI_2_ was omitted from, the solution throughout the incubation period. The amount of loaded [^3^H]‐NE released into the media was counted using a Beckman‐Coulter LS6500 Scintillation Counter. Fractional release is defined as the amount of [^3^H]‐NE released over the amount of [^3^H]‐NE in the tissue at that time point. The net fractional release is the release in the presence of stimulus (glutmate or K^+^) minus the mean of the three basal release wells immediately preceding the stimulus condition.

## 5 BEHAVIORAL EXPERIMENTS

### 5.1 Novel object recognition task

The animals were individually habituated to a Plexiglass enclosure (dimensions: 16"w × 16"d × 16"h) for 5 min/day for 5 days. On Day 6 (training phase), two identical objects (A + A) were placed diagonally in the arena and affixed to the floor. All objects were of varying shapes and sizes, ranging between 3 and 4 inches. The rat was placed in the center of the arena and allowed to explore A + A in two 5 min trials, each with a 5‐min inter‐trial interval. After 24 h, one object was replaced by a new one (A + B), and the animal returned to the experimental apparatus and was allowed to explore the novel and familiar objects for 5 min (test phase). After each trial, the arena and the objects were cleaned with 70% ethanol to minimized olfactory cues. Videos were recorded by a camera positioned above the apparatus and analyzed by a blind observer. Exploration of an object was defined as the rat's nose being within 1 cm of and oriented toward the object, sniffing at, or otherwise closely attending to the object. A DI value between −1 and +1 was calculated as the difference in time exploring the novel and familiar object, expressed as the ratio of the total time spent exploring both objects ([TN‐TF]/[TN + TF]). Rats showing a total exploration time of <5 s during the training phase were excluded.

### 5.2 Barnes maze

For this set of behavioral experiments, a new cohort of aged Fischer 344 rats were used than those previously tested in the NOR task. Rats were trained in the task, as previously described (Negrón‐Oyarzo et al., 2015). Briefly, in each session, rats explored an elevated circular platform with 18 equidistant holes, in which one served as the escape hole. To encourage the animal to seek out the escape hole, a bright light aversive stimulus was applied above the surface of the maze. Extra‐maze cues were placed around the room as reference cues to learn the position of the target hole. Each rat received four daily trials for four consecutive days for the acquisition phase. Primary latency was measured as the time to the target hole and primary errors were defined as nose‐poke in nonescape holes (Gawel et al., 2019).

### 5.3 Golgi staining and confocal imaging

Following perfusion with ice‐cold phosphate‐buffered saline, whole brains were stained with the FD Rapid Golgi Stain Kit (FD NeuroTechnologies, #PK401). Sections from the prefrontal cortex (Bregma A/P + 2.0 mm) and dorsal hippocampal formation (A/P‐3.3 mm) were taken at approximately 100 μm on a Leica VT1200S series vibratome (Leica Biosystems) and mounted on gelatin‐coated slides. After air‐drying at room temperature overnight, the sections were stained for 10 min in staining solutions, dehydrated by sequentially ethanol washes, and cleared in Hemo‐DE xylene substitute (Electron Microscopy Sciences Inc., #23412‐01), and cover slipped with Permount resinous media (Thermo Fisher Scientific Inc., SP15‐100). Sections were imaged on a Leica confocal microscope with parameters as previously described (Speidell et al., 2020). For imaging, regions with spines from the basal dendrites of layer II/III pyramidal neurons (prelimbic cortex) or granule cells of the DG (hippocampus), which lay approximately 100–150 μm from the neuron soma, were selected. Blinded observers used the companion Leica LASX software to assess spine density (spines/10 μm) and spine height and width (in microns) in image stacks of these regions. These spines were further classified into one of the seven spine subtypes as described from previous morphological studies (Risher et al., 2014).

### 5.4 Quantification and statistical analysis

NE release data were analyzed with a simple Student's t test to compare two groups, and for more than two groups, a mixed effect ANOVA was followed by Dunnett's multiple comparisons tests. EC_50_ and E_max_ values were obtained by fitting the values to a sigmoidal dose–response curve with a variable slope identical to the three‐parameter logistic equation (Top, Bottom, and Log EC_50_). For the behavioral studies, data were analyzed using one‐way, two‐way, and three‐way ANOVA with Tukey's post hoc test or Fischer's LSD test corrected with Holm–Sidak for multiple comparisons. Dendritic spine density was analyzed using the MIXED procedure in SPSS version 29 (IBM). A marginal model was employed with age and drug as between‐subject factors and cell as a within‐subject factor. Data were analyzed and plotted using GraphPad Prism software v. 9.0 and Microsoft Excel software. An alpha level of *p* < 0.05 was the criterion used to determine statistical significance.

## AUTHOR CONTRIBUTIONS

Serena Scognamiglio, Yousef M. Aljohani, Thao T. Olson, Ghazaul Dezfuli, and Kenneth J. Kellar conceptualized and designed the research. Serena Scognamiglio, Yousef M. Aljohani, Thao T. Olson, and Ghazaul Dezfuli performed the research. Serena Scognamiglio, Yousef M. Aljohani, Thao T. Olson, Patrick A. Forcelli, and Ghazaul Dezfuli analyzed the data. Serena Scognamiglio, Yousef M. Aljohani, Ghazaul Dezfuli, and Kenneth J. Kellar wrote the paper.

## FUNDING INFORMATION

Georgetown University Partner's in Research Philanthropic Grant.

## CONFLICT OF INTEREST STATEMENT

The authors have declared no competing interests.

## INCLUSION AND DIVERSITY

We support inclusive, diverse, and equitable conduct of research.

## Supporting information


Appendix S1.

## Data Availability

The data that support the findings of this study (including raw images), and any additional information are available upon request from the lead contact Ghazaul Dezfuli (gd96@georgetown.edu).
