# Supplementary material for: Restoration of norepinephrine release, cognitive performance, and dendritic spines by amphetamine in aged rat brain
Source: Aging Cell. 2024 Feb 8;23(4):e14087. doi: 10.1111/acel.14087 (PMC11019150; doi:10.1111/acel.14087)
Supplement: Supplementary file 1 — Appendix S1. [file ACEL-23-e14087-s001.docx]

**Combined SI appendix & datasets**

**
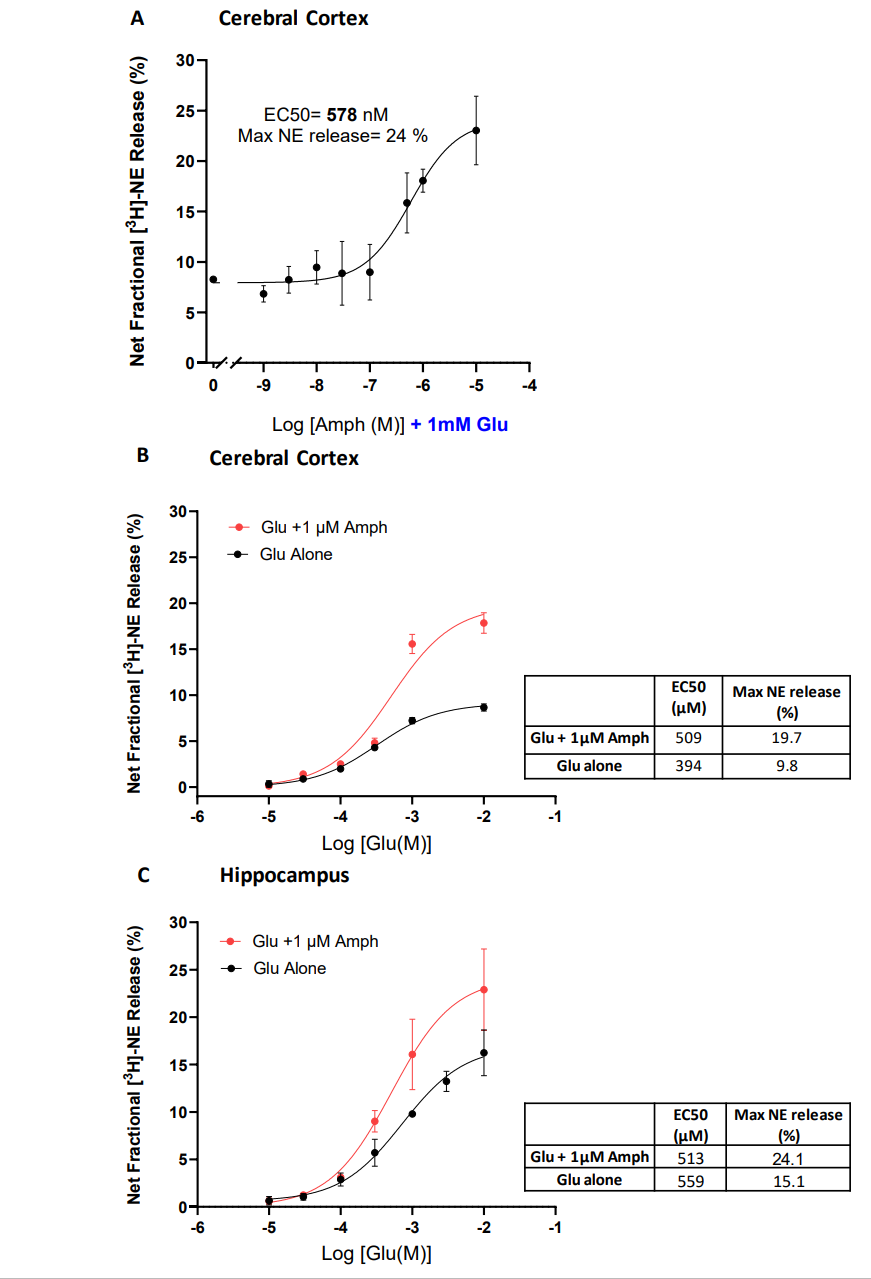
**

**Figure S1. *Effects of D-amphetamine on the glutamate-stimulated release of [^3^H]­-norepinephrine in the cerebral cortex and hippocampus from naïve young* Fischer 344 *rats.* (A)** Concentration-response curve of D-amphetamine in the presence of 1mM glutamate in the cerebral cortex. The glutamate concentration-response curve in the absence (black curve; n=2) and presence (red curve; n=3) of 1 µM D-amphetamine sulfate in **(B)** the cerebral cortex and **(C)** the hippocampus. Each data point is the mean (± SEM) of the net fractional release of 2-3 independent experiments. The glutamate curves shown in **(B)** and (**C)** are from the same data shown in

**Figure 1**.

**Figure S2. *Summary pie chart indicating the quantification of dendritic spine morphology after treatment with chronic amphetamine.*** Data are presented as percentages of mature spines (stubby, branched, mushroom, & SHP) vs. immature spines (filopodia, thin, & long thin). Data are displayed as mean; n=5-6/group.**
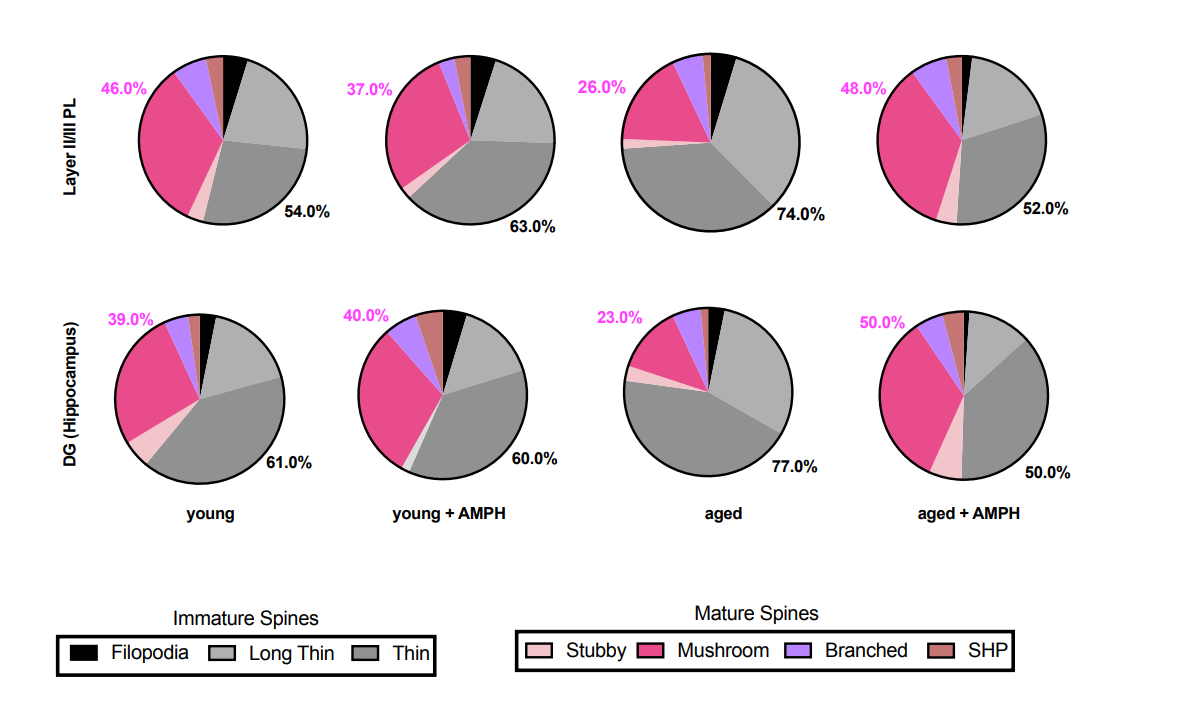
**
